# Teaching meta-analysis using MetaLight

**DOI:** 10.1186/1756-0500-5-571

**Published:** 2012-10-18

**Authors:** James Thomas, Sergio Graziosi, Steve Higgins, Robert Coe, Carole Torgerson, Mark Newman

**Affiliations:** 1 EPPI-Centre, Social Science Research Unit, Institute of Education, University of London, London, UK; 2 School of Education, University of Durham, Durham, UK

**Keywords:** Meta-analysis, Research synthesis, Systematic review, Teaching, Teaching resource, Software

## Abstract

**Background:**

Meta-analysis is a statistical method for combining the results of primary studies. It is often used in systematic reviews and is increasingly a method and topic that appears in student dissertations. MetaLight is a freely available software application that runs simple meta-analyses and contains specific functionality to facilitate the teaching and learning of meta-analysis. While there are many courses and resources for meta-analysis available and numerous software applications to run meta-analyses, there are few pieces of software which are aimed specifically at helping those teaching and learning meta-analysis. Valuable teaching time can be spent learning the mechanics of a new software application, rather than on the principles and practices of meta-analysis.

**Findings:**

We discuss ways in which the MetaLight tool can be used to present some of the main issues involved in undertaking and interpreting a meta-analysis.

**Conclusions:**

While there are many software tools available for conducting meta-analysis, in the context of a teaching programme such software can require expenditure both in terms of money and in terms of the time it takes to learn how to use it. MetaLight was developed specifically as a tool to facilitate the teaching and learning of meta-analysis and we have presented here some of the ways it might be used in a training situation.

## Findings

### Background

Meta-analysis is a statistical method for combining the results of primary studies. It is often used in systematic reviews and is increasingly a method and topic that appears in student dissertations. There are many courses and resources for meta-analysis available online, such as: the Cochrane open learning materials; two ESRC RDI courses^a^; Michael Borenstein’s course on Statistics.com^b^; reusable learning objects [1]^c^; and the EPPI-Centre’s MSc module on synthesis^d^. This paper, and the software it describes, is a contribution to these resources; aiming to support the teaching of meta-analysis and its use in student learning.

### Results and discussion

#### MetaLight functionality

MetaLight [2]^e^ is a lightweight application that aims to support the teaching of meta-analysis. Its interface has been designed to develop understanding of: the relationship between effect sizes and their appearance on the forest plot; the impact on results of selecting a fixed or random effects model; and how funnel plots are constructed. It also contains a forest plot exercise feature that enables students to ‘draw’ a forest plot based on descriptive text, rather than being presented with one to interpret.

While MetaLight supports a wide range of analytical functions, it is intended primarily as a tool for teaching and learning; for use in the classroom and for conducting student assignments. Other tools for meta-analysis that support advanced analyses such as meta-regression and Bayesian meta-analysis have been recently summarised elsewhere [3].

**Figure 1:**
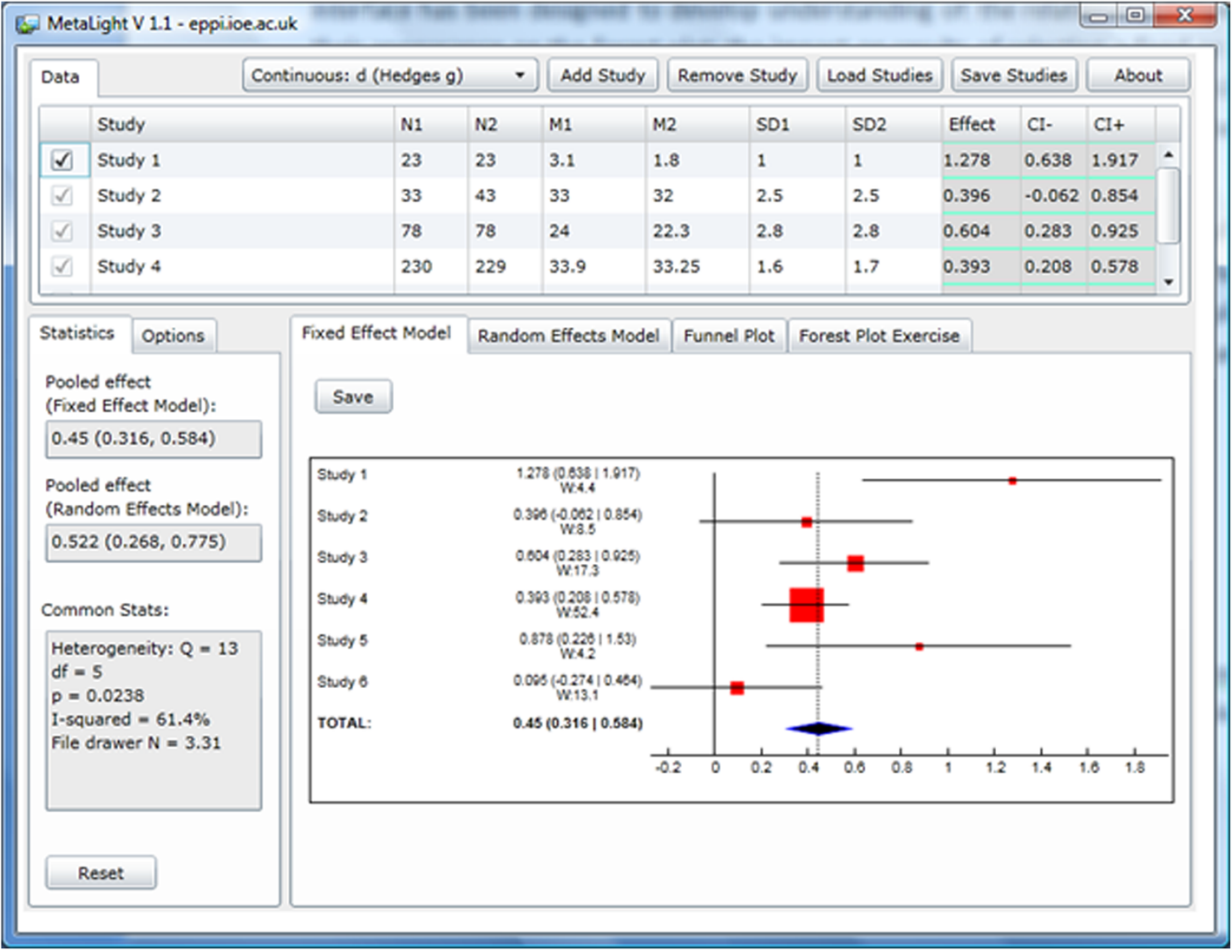
The MetaLight application.

The opening screen of MetaLight is split into three main areas as displayed in Figure 1. Across the top of the window is a grid that lists all the studies in the meta-analysis along with statistics that enable effect sizes to be calculated for each study. Occupying the largest area of the screen is a panel that displays the results of the meta-analysis in the form of forest and funnel plots; its pooled effect sizes and heterogeneity statistics are displayed to the left of this.

The program contains what might be considered to be standard meta-analytic functions. It calculates the following effect sizes from either continuous or binary data assuming a standard two group (typically intervention and control) study design: Hedges g, r (correlation), mean difference, odds ratio, risk ratio, risk difference. Hedges g and the mean difference are calculated based on the sample sizes of each group, their means and standard deviations (see Borenstein et al. 2009 [4] for details of the formulae used in MetaLight). An r effect size is taken as entered, using its sample size to estimate its standard error. Binary effect sizes are calculated using a standard 2 × 2 table that summarises ‘events’ and ‘no events’ in the each group. MetaLight contains a set of data for each outcome type (see below) and data can also be saved and loaded from a text file.

Effect sizes are pooled using fixed and random effects models with the Heterogeneity statistic Q, I^2^, and ‘file drawer N’ given on the left. The meta-analysis is displayed graphically in two forest plots (fixed and random effects models) and funnel plot.

MetaLight can thus be used to conduct straightforward meta-analyses and the forest plots can be exported for insertion in, for example, student assignments.

**Figure 2:**
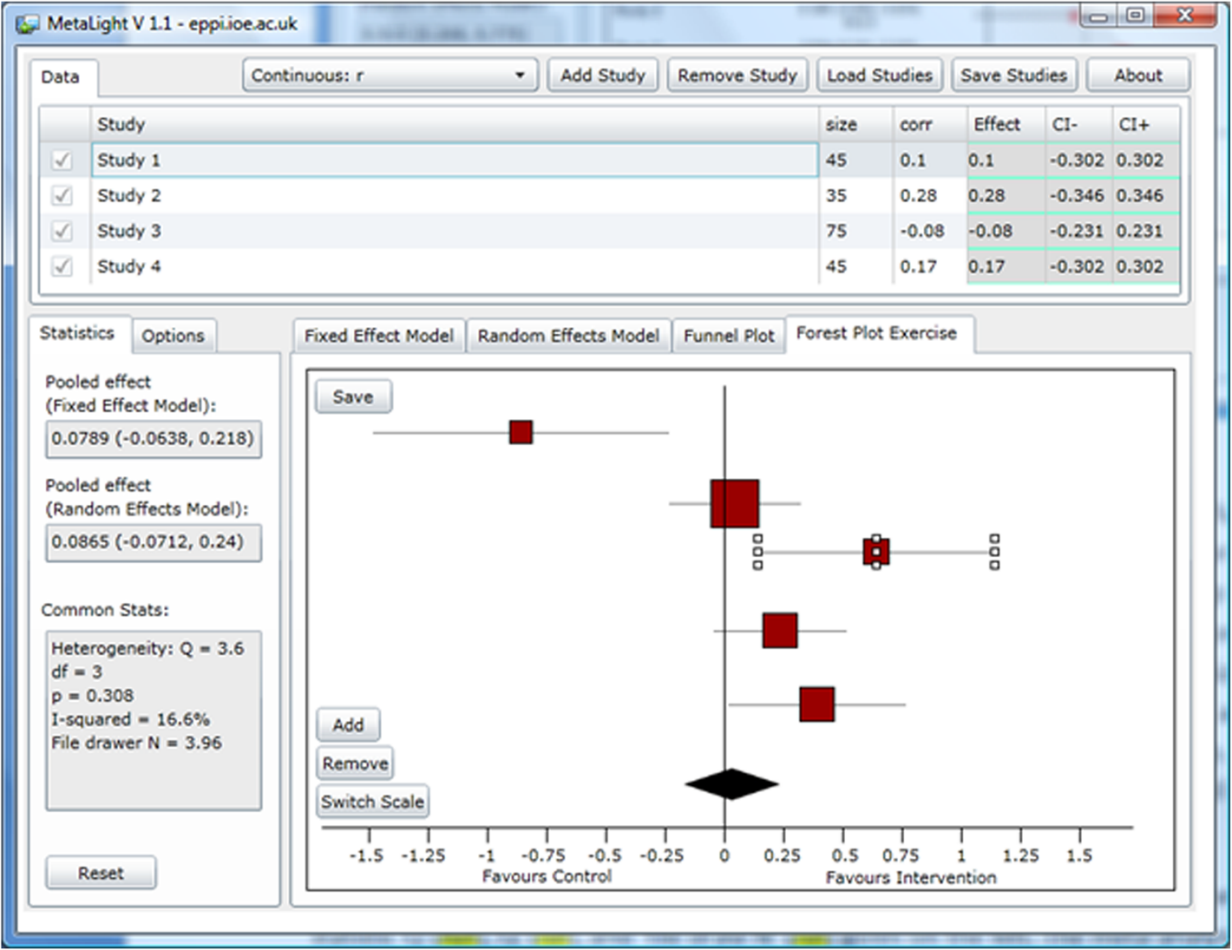
The forest plot exercise tab.

In addition to the above functionality, the forest plot pane has a tab which displays a forest plot that can be manipulated by students (Figure 2). Point estimates can be dragged and dropped around the plot; the size of the central square can be resized; and confidence intervals can be increased and decreased.

#### Teaching meta-analysis using MetaLight

MetaLight has been designed primarily as a teaching aid. It is a Silverlight application that can be run in a web browser and thus used in a PC lab environment, requiring only that the Silverlight plug-in is installed and internet access is available. There are two main ways of using MetaLight: as a visual aid during lectures, and as a tool to be used during exercises and assignments. While the MetaLight application has been extended to support a range of types of effect size, the teaching materials presented here focus on the standardised mean difference.

If students can view the MetaLight application (e.g. via a projector or software such as Elluminate that enable users to ‘share’ desktop displays), a lecturer can talk students through the relationship between individual studies and the resulting forest plot and heterogeneity statistics. The ‘anatomy’ of a forest plot can be discussed, including such issues as the visual recognition of heterogeneity through non-overlapping confidence intervals and understanding the importance of different studies as represented by the size of the square that represents their effect size.

MetaLight loads with a set of data ready to use. This dataset has been designed to facilitate the exploration of subjects such as heterogeneity and statistical models and is the focus of the example worksheet given in Additional file 1: Appendix A. The following sections outline how MetaLight might be used to illustrate some of the key issues in conducting a meta-analysis and interpreting a forest plot. We begin with the primary studies that make up the meta-analysis, covering how they are represented on the forest plot and how differences in, for example sample size, can be established visually. We then move on to the meta-analysis itself: how the pooled effect size is represented and differences between statistical models. Publication bias and funnel plots are the final issue to be tackled, before some example student exercises are presented.

The examples mentioned in the text assume that the MetaLight program has been opened and the default data have not been changed.

##### Understanding how individual studies are represented on the forest plot

The forest plot is an unusual graph for those who have not seen one before, as it contains a great deal of information that requires an understanding of how each individual study is represented. Important concepts (which are *italicised*) to understand are as follows.

Each *row* in the forest plot represents one *study*, with a short description for identification given on the left (often in the format: author (year of publication)). The effect size of each study is represented by a *red box*, the size of which is related to the relative *weight* accorded to that study in the analysis (larger boxes catch the reader’s attention deliberately, since their results are given more weight). Larger studies are usually given greater weight and so appear visually larger than smaller ones. In the example data, Study 4 is clearly the largest study, contrasting with Study 5, which has a much smaller box to represent its effect size estimate. Lines extend either side of the effect size representing the *confidence intervals* around it. Again, attention can be drawn to the difference between large and small studies, with smaller studies tending to have wider confidence intervals – reflecting a greater degree of uncertainty about precisely where their ‘true’ result is. Using the default data, attention can be drawn to the wider confidence intervals around Study 5, compared to the much tighter intervals around Study 4. The vertical line which meets the x-axis at 0 (or 1 for some types of effect size) is the *line of no effect*. Usually studies which are plotted to the right of the line have a positive outcome and those to the left a negative one; but this does depend on the type of effect measure being used. Importantly, outcomes with confidence intervals which cross the line of no effect are not *statistically significant*; this is clear, since the confidence interval encompasses both positive and negative results. In the example data, the confidence intervals around Studies 2 and 6 span this *line of no effect* and their results are thus not statistically significant; all the other studies are reporting statistically significant findings. The *relative magnitude* of effect sizes between studies can be compared with one another with those further from the line of no effect being larger than those closest to it.

The forest plot is updated as soon as data relating to a study have changed, so the lecturer is able to demonstrate the impact of different study parameters on individual studies’ effect sizes and the overall results of the meta-analysis. Changing the size (number of participants) in a study has very little bearing on its effect size, but does change its standard error noticeably – which can be seen in the length of its confidence intervals and the size of the square that represents how much weight it is accorded in the analysis. (The fact that a study’s effect size is marginally related to the number of participants it contains may lead to a discussion as to why this should be and whether it matters.) A good example of this using the sample data is to reduce the size of the two groups (N1 and N2) in Study 4 to 23 and 22 (rather than 230 and 229). The effect size estimate remains almost the same, but the confidence intervals widen significantly – to the point that they cross the line of no effect, making the results no longer statistically significant.

Changing one or more of the means changes the magnitude of the effect size and possibly also its direction. Lecturers can demonstrate how the square representing the effect size moves horizontally when the mean(s) it is based on change, but that its confidence intervals remain much the same size – as does the size of its red square (i.e. its weight in the analysis). (For example, reducing the comparison mean (M2) in Study 1 from 2.8 to 1.8 moves the study to the left on the forest plot, whilst keeping its confidence intervals the same width.)

Changing the standard deviation(s) has results that might require discussion and further elaboration. One might expect that changing the variance of individual studies would result in changes to the confidence intervals around their effect sizes – as confidence intervals are related to a study’s variance – and this is indeed the case. It is, however, difficult to demonstrate this, because changing the variance also changes the *effect size* itself. (This can be demonstrated using the sample data by changing the standard deviation of the experimental group (SD1) in Study 1 to 2, thus reducing the effect size estimate.) This is, of course, by design, but there are interesting consequences that might be worthy of discussion. For example, suppose we had a series of studies that all measured depression using the same tool. We could presumably dispense with the need to standardise results and simply meta-analyse mean differences (MetaLight can be used to demonstrate this). What if, however, the standard deviations differed between studies? It can be seen that studies may have identical numbers of participants and means, but if they have different standard deviations, will have different standardised mean differences. Again, this can be demonstrated visually. What would be the most valid method of combining their results? This may lead into a discussion about methods for interpreting the standardised mean difference by, for example, considering the proportion of the control group that would be above or below the mean of the intervention group [5].

##### Understanding the pooled effect size and the contribution of individual studies

Once the effect sizes of individual studies have been explained, the *diamond* representing their pooled aggregation is a small additional step. It has no red box, and no relative weight, as it is a qualitatively different piece of information. Again, its position relative to the x axis represents its *magnitude and direction*, its confidence intervals represented by its right and left points, and statistical significance can be established quickly by seeing whether they cross the line of no effect. However, while these are relatively simple concepts to convey, the relationship between individual studies and their pooled result is worthy of exploration, as is the difference between the *statistical models* for pooling effect sizes. In particular, the *relative weights* of studies using different models is worth discussing, as is the *greater uncertainty* around the pooled effect size observed in the random effects model (compared with the fixed effect model).

As described above, the instant connection between data entry and the forest plot can be used to demonstrate the impact of changing the number of participants in a study. Notably, even though changing the number of participants usually makes very little difference to the effect size of the individual study, such a change *can* change the pooled effect size of the meta-analysis, as the weight given to the study’s effect size changes in relation to the number of participants it has. In the same way, changes to a study’s effect size can have more or less effect on the pooled effect size in the meta-analysis depending on the amount of weight given to each study. (For example, when reducing the sample size of Study 4 by a factor of 10 in the sample data (by reducing N1 to 23 and N2 to 22), the pooled effect size increases, because the weight given to that formerly large study has reduced.)

**Figure 3:**
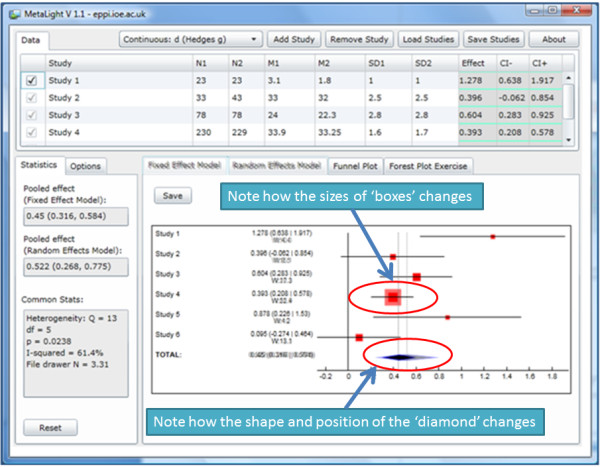
Observing differences between models.

Forest plots depicting the results of a meta-analysis using fixed and random effects models are shown in neighbouring tabs and are positioned at identical points on the screen. This facilitates the rapid switching between graphs which highlights differences between them. The size of the squares that represent the weight that each study received can be seen as increasing or decreasing between the models; and changes in the pooled effect size – along with its confidence intervals – are also apparent. Figure 3 shows this, containing two screenshots superimposed over one another: the weight given to Study 4 changes markedly, as does the position of the pooled effect size and the size of its confidence intervals. An instructor can demonstrate this by clicking between the ‘fixed effect model’ tab and the ‘random effects model’ tab. The effect size estimates of the studies remain in the same place, but the sizes of their red boxes change, showing how they are weighted differently in the two analyses; the pooled effect size moves, and the shape of its diamond changes, showing how it has been affected by the changes in weight accorded to different studies.

The fact that the random effects model (with a heterogeneous set of outcomes) gives considerably greater weight to smaller studies is a topic for discussion. While the statistical rationale for this can be clarified, people will have differing views as to whether or not giving greater weight to smaller studies is desirable; some might say that this merely fixes the bias towards large studies present in the fixed effect model.

##### Publication bias

Previous empirical work has shown that studies are more likely to be published if they have positive results [6]; small studies with negative or equivocal results are less likely to be published. While discussion might focus on the many reasons that there may be for bias to affect published outputs, the key point is that this bias is *systematic*: it may therefore lead to biased conclusions in a meta-analysis. While there is little that may be done practically about publication bias in a meta-analysis (though see Duval & Tweedie 2000a and b [7,8]), it is good practice to examine a set of studies to see whether publication bias may be detected.

The visual means of examining publication bias is through a funnel plot (displayed under the ‘funnel plot’ tab), which typically consists of the study effect sizes on one axis and the standard error of the effect sizes on the other axis. The key concept in the funnel plot, where studies are plotted according to their effect size (x – axis) and variance (usually standard error on the y – axis), is that the graph should take on the appearance of an *inverted funnel*, with the larger studies, with small variances clustered towards the top, and smaller studies, with their greater variance, more scattered towards the bottom. Since publication bias affects smaller, negative, studies more than larger or positive ones, the theory is that publication bias (when present) will result in a ‘hole’ in the funnel plot in the space where smaller, negative, studies would be plotted – in the bottom left of the graph. The problem is that many meta-analyses contain *too few* studies to form a recognisable funnel, and that an asymmetrical funnel plot may not necessarily be due to publication bias at all. The sample data have been so selected so as to stimulate discussion about this issue. One may take the view that the data may be affected by publication bias because of the absence of studies towards the bottom left of the graphic. On the other hand, there are very few studies in the analysis, so it is difficult to draw any conclusions from the plot on its own.

**Figure 4:**
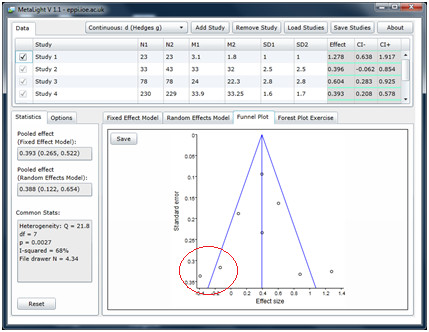
Publication bias exercise.

Interaction between the grid of studies and the funnel plot is also helpful for teaching. In this case, the lecturer can increase the number of participants incrementally and show how this gradually ‘moves’ a study higher on the y axis without changing its position on the x axis. This should help to reinforce earlier discussion about the relationship between the size of the study and its variance, as opposed to its result, which is mostly independent of its size. A useful (and challenging) exercise is to ask students to add in the ‘missing studies’ in the default MetaLight dataset. As well as understanding how a funnel plot works, this task again requires engagement with effect sizes and the relative size of studies. Figure 4 shows the addition of two studies in the ‘gap’ which may be due to publication bias.

##### Student exercises

As MetaLight is freely available online and can be accessed quickly in a PC lab setting, it can also be used for group or individual exercises. An example worksheet can be found in Additional file 1: Appendix A. One approach to using this worksheet in a class setting is to present and discuss each issue in turn (running a meta-analysis, heterogeneity, statistical models, publication bias and sensitivity analysis) and then complete the worksheet individually or in pairs; answers can then be discussed in a whole group. The ‘construction’ of a forest plot works well as a group exercise, with students directing where effect sizes should be located, how long their confidence intervals should be, and how large the red boxes should be. The lecturer positions each graphical component and checks that the students are in agreement as to their final locations and what the implications are.

### Conclusions

While there are many software tools available for conducting meta-analysis, in the context of a teaching programme such software can require expenditure both in terms of money and in terms of the time it takes to learn how to use it. MetaLight was developed specifically as a tool to facilitate the teaching and learning of meta-analysis and we have presented here some of the ways it might be used in a training siutation.

### Technical details

MetaLight requires the Silverlight plug-in^f^ that can run in a web browser as well as enabling the application to run ‘out of browser’ if needed. MetaLight was developed to support a series of ESRC-funded workshops to develop capacity in meta-analysis in education in the UK. This project, running from April 2008 to March 2009 aimed to: develop understanding of meta-analysis to enable more informed critique; foster a need for its considered application in educational research; and to meet a need for the further development of innovative methods and techniques in education. The teaching resources from this project are available online at: http://www.dur.ac.uk/education/meta-ed/.

## Availability and requirements

Project name: MetaLight

Project home page: http://eppi.ioe.ac.uk/cms/Default.aspx?tabid=3086.

Operating system(s): Windows (any); OS X (possibly Linux etc., depending on Mono support).

Programming language: C#.

Other requirements: Microsoft Silverlight browser plugin

License: MetaLight is free for use in any environment, including but not necessarily limited to: personal, academic, commercial, government, business, non-profit, and for-profit.

Any restrictions to use by non-academics: none.

## Endnotes

^a^http://www.dur.ac.uk/education/meta-ed/ and http://www.self.ox.ac.uk/metamenu.htm.

^b^http://www.statistics.com/course-catalog/meta/.

^c^http://www.nottingham.ac.uk/nursing/sonet/rlos/ebp/meta-analysis2/.

^d^http://eppi.ioe.ac.uk/cms/Default.aspx?tabid=696.

^e^Freely available at: http://eppi.ioe.ac.uk/free-tools/meta-analysis/.

^f^http://www.silverlight.net.

## Competing interests

The authors declare that they have no competing interests.

## Authors’ contributions

All authors contributed to the design of the software and to the drafting of this paper. SG undertook much of the programming with some code written by JT. JT, SH, RC, CT and MN were all involved in drafting the student exercises. All authors read and approved the final manuscript.

## Supplementary Material

Additional file 1 **Appendix A.** Worksheet. Click here for file
