# Supplementary material for: Teaching meta-analysis using MetaLight
Source: BMC Res Notes. 2012 Oct 18;5:571. doi: 10.1186/1756-0500-5-571 (PMC3532381; doi:10.1186/1756-0500-5-571)
Supplement: Additional file 1 — Appendix A. Worksheet. [file 1756-0500-5-571-S1.docx]

# Appendix A: Worksheet

# Running and exploring a meta-analysis

## a) Running a meta-analysis

Load MetaLight and, if you have changed any data, Reset to default values by using the ‘Reset’ button at the bottom left.

1) Does the meta-analysis suggest the interventions in this review had a beneficial effect? What does the forest plot show on the ‘Fixed effect model’?

2) For which studies does the 95% confidence interval *exclude* the possibility of no effect or a negative effect?

3) What is the pooled effect size (for the fixed effect model) and confidence intervals for this? How is this represented in the meta-analysis?

4) What do the different sizes of the red squares indicate?

## b) Heterogeneity

Have a look at the information on the left hand side next to the forest plots.

1) How much heterogeneity is there?

i) Does the forest plot suggest there is heterogeneity?

ii) What does the heterogeneity statistic show? (Q)

iii) How much heterogeneity does I-squared suggest there is?

2) Do you think there is too much heterogeneity to combine this set of studies statistically?

3) Looking at the forest plot, which study is the most obvious outlier?

4) Uncheck the outlier study (uncheck the tick at the left) and run the analysis again. What is the effect of removing this study on the fixed effect analysis?

## c) Models: fixed effect and random effects

Include the outlier again (check the box) and click ‘calculate’, then look at the ‘Random effects model.

1) Looking at the results of your last analysis (c), what are the differences between the results based on the fixed effect, as opposed to random effects, model? How do the pooled effect sizes compare? What about the differences in the forest plots?

2) Which model would be the most appropriate to use with this dataset and why?

## d) Publication bias

1) Look at the funnel plot for your meta-analysis. What are the implications of this funnel plot for your meta-analysis?

2) See if you can add two studies to the analysis (using the ‘Add study’ button) to simulate studies which might not have been found due to publication bias (i.e. which will appear in the bottom left corner of the funnel plot). Estimate the *effect size*  and sample size you will need for the studies to appear in this part of the chart.

When you are satisfied that the funnel plot is more or less symmetrical click on the ‘forest plot’ tab. How has this changed the meta-analysis?

Do you think it is likely there was publication bias?

## e) Sensitivity analysis

1) Conduct a sensitivity analysis using the default data (click ‘Reset’ to get back original values if you have changed them). How reliant are your conclusions on the results of one or two studies?

2) Increase the sample size of study 1 to approximately 100 in each group. How does this affect your sensitivity analysis?

## f) Constructing a forest plot

Run MetaLight and click the ‘Forest Plot Exercise’ tab.

Read the following description of a meta-analysis and ‘sketch’ its forest plot in MetaLight. The positioning of effect sizes and confidence intervals does not need to be exact; the main purpose of this exercise is being able to relate the descriptions of the studies with one another and so understand the relative positions of their confidence intervals and sizes of effect size ‘boxes’ (the red squares that show the magnitude of the effect size).

Summary statistics for the meta-analysis were as follows:

- Its pooled effect size was 0.241 (0.128, 0.355)
  Heterogeneity statistic Q = 12.5, df = 4, p = 0.0139, I-squared = 68%
- Study 1 was a small study which, while not statistically significant, suggested the intervention had a small negative effect.
- Study 2 was the most positive about the intervention, suggesting it had an effect size of at least 0.25 and may have been as high as 1.15. It was also a relatively small study.
- Study 3 was a medium sized study that suggested the intervention had a moderate positive effect. This was statistically significant.
- Study 4 had the same effect size as Study 3, but as it was much smaller its confidence interval included the possibility that the intervention may have had a small negative effect.
- Study 5 was much the largest study, being accorded 50% of the weight of all the studies. Its effect size was less than half the size of studies 3 and 4, and while the direction of effect was positive, this was not statistically significant.
